# On the Quantum Chemical Nature of Lead(II) “Lone Pair”

**DOI:** 10.3390/molecules27010027

**Published:** 2021-12-22

**Authors:** Christophe Gourlaouen, Jean-Philip Piquemal

**Affiliations:** 1 Laboratoire de Chimie Quantique, UMR7177 CNRS et Université de Strasbourg, 67000 Strasbourg, France; 2 Laboratoire de Chimie Théorique, Sorbonne Université, UMR7616 CNRS, 75005 Paris, France; 3 Institut Universitaire de France, 75005 Paris, France; 4 Department of Biomedical Engineering, The University of Texas at Austin, Austin, TX 78712, USA

**Keywords:** Pb^2+^, lone pair, top logical analysis, quantum chemistry, lead complexes

## Abstract

We study the quantum chemical nature of the Lead(II) valence basins, sometimes called the lead “lone pair”. Using various chemical interpretation tools, such as molecular orbital analysis, natural bond orbitals (NBO), natural population analysis (NPA) and electron localization function (ELF) topological analysis, we study a variety of Lead(II) complexes. A careful analysis of the results shows that the optimal structures of the lead complexes are only governed by the 6s and 6p subshells, whereas no involvement of the 5d orbitals is found. Similarly, we do not find any significant contribution of the 6d. Therefore, the Pb(II) complexation with its ligand can be explained through the interaction of the 6s^2^ electrons and the accepting 6p orbitals. We detail the potential structural and dynamical consequences of such electronic structure organization of the Pb (II) valence domain.

## 1. Introduction

Since antiquity, Lead has been considered a metal of prime interest. While we now know more about its toxicity, many industrial applications still use it and the environmental impacts are significant [1]. For this reason, beyond the constant interest in a deeper understanding of the synthetic chemistry and associated properties of Pb(II) compounds, strong motivation exists towards designing molecules capable of selectively chelating Pb(II) towards applications in medicine [2,3] or in the environment [4]. Although substantial efforts have been dedicated towards the analysis of the solid-state properties of lead-containing materials [5,6], an in-depth comprehension of the Pb(II) coordination at a molecular level remains lacking [7,8,9,10], and theoretical computations have been shown to be important. Indeed, since the first calculation by Shimoni-Livni [11], which highlighted the possibility of holodirected and hemidirected geometries for complexes where Pb(II) is coordinated to four ligands, the nature of the lacuna in low-coordinated lead complexes has been discussed. Its origin has been linked to the orbitals involved in the complexation. The electronic configuration of the Lead(II) cation (i.e., Pb^2+^) is [Xe] 4f^14^ 5d^10^ 6s^2^ 6p^0^ 6d^0^. The highest occupied orbital is the full 6s and the lowest unoccupied is the empty 6p. The s nature of the HOMO orbital theoretically does not allow any directionality of the electronic pair, so the origin of the lacuna is still under discussion. The first hypothesis was that the s electronic pair was polarized by the 6p orbital, which would enable the creation of a hybrid “sp” lone pair. However, the nature of the orbital and natural bond orbital (NBO) analysis show that the p character of the electronic pair is very weak, being approximately only about 5% [11,12]. Understanding this bonding pattern is very important as the tendency of the Lead(II) cation to exhibit a lacuna in its coordination sphere seems strongly linked to the cation’s toxicity (i.e., the well-known lead poisoning). We have shown in a previous paper [13] that what we called the “lead lone pair” was at the origin of the perturbation of proteins through its ability to restructure the metallic coordination sphere.

In another previous work dedicated to a series of lead ligands, we studied, by means of electron localization function (ELF) topological analysis, the evolution of the volume and the density of the Lead(II) valence basin [14]. We obtained a population larger than 2 electrons showing that charge transfer could play a role in the emergence of a directional valence basin. However, all calculations were performed using large core pseudopotentials in which the 5d electrons were merged in the core so that only the 6s electrons remained. Therefore, we were not able to investigate the possible role of these low-lying 5d orbitals. In this contribution, we propose to return to this subject using all the available analytic tools to investigate the nature of the Lead(II) cation valence basin cation in order to determine the orbital involved within it. Then, we will extend our study to the flexibility of the hemidirected structures of lead complexes.

## 2. Computational Details

An initial set of calculations were performed using the TURBOMOLE package [15] at the MP2 level of theory using a def2-TZVP basis set [16] (with associated small-core pseudopotential for Pb and I atoms) in the gas phase. The quantum chemical results were analyzed by means of natural population analyses (NPA) and natural bonding orbital (NBO) analyses [17,18]. To perform the topological analyses, we extracted the TURBOMOLE optimized geometries and computed the wavefunction with the GAUSSIAN09 version D01 package [19] within the B3LYP [20] formalism with the same basis set.

On the basis of this wavefunction, electron localization function (ELF) calculations and the topological analysis of the ELF functions, together with specific integrations, were performed using the TopMod package [21,22,23,24,25,26,27,28]. Here, we simply recall that, within the framework of the topological analysis of the ELF function, space is partitioned into basins, each of them having a chemical meaning. Such basins are classified as: (i) core basins surrounding nuclei, (ii) valence basins characterized by their synaptic order [21]. Further details can be found in the above-mentioned references.

It has been shown that it is possible to extend the ELF approach to pseudopotential approaches [29]. Using small-core pseudopotentials provides the so-called semi-external cores and allows the determination of the synapticity of well-defined valence basins [30]. Using large-core pseudopotentials preserves the number and the properties of valence basins. Only small-core pseudopotentials have been used. In the present contribution, we focus on V(Pb), the valence monosynaptic basin associated with the valence electrons of Pb(II). For a given complex [Pb(II)L_n_]^q^, we use the following notations: V(Pb) is the ELF basin defined previously; N(Pb) and ω(Pb) are, respectively, the population and the volume associated with this basin. The final parameter is the distance d(Pb) between the ELF attractor of the lead valence basin V(Pb) and the lead cation itself.

A second set of geometry optimizations were performed with GAUSSIAN at the DFT level of theory with B3LYP and the more recent ωB97XD [31] functionals on the [Pb(CN)_3_(HCl)]^−^, [Pb(CN)_3_(ClH)]^−^, [Pb(CO)_3_(HCl)]^2+^ and [Pb(CO)_3_(ClH)]^2+^ complexes. To explore the nature of the interaction between the Pb(II) complexes and the extra HCl probe molecule, a non-covalent interactions (NCI) study was performed on the basis of the optimized geometry wavefunction using the NCIPLOT package [32] (nciplot3 version). For some complexes, we also searched for the transition state to compute the interconversion barriers. All the provided energies are Gibbs free energies as obtained from GAUSSIAN frequency calculation at 298 K.

We would like to point out that, for ligands in the first coordination sphere, there are very few observed differences for the bond lengths between the different methods. In practice, the values are close to those already published, as illustrated in Table S1 (Supplementary Materials).

## 3. Results and Discussion

We performed full geometry optimization on several lead complexes. The first was the [Pb(CO)_n_]^2+^ series, which is easy to analyze thanks to the ligand rigidity. Secondly, we focused on complexes with various organic or inorganic ligands. These complexes represent a small sample of the potentially existing lead complexes; however, they have all been studied and represent a variety of coordination modes and conformations. On all these complexes, we studied the involvement of lead orbitals in the coordination and in the complex topology.

The Pb(II) electronic configuration is [Xe] 4f^14^ 5d^10^ 6s^2^ 6p^0^. The 4f shells are very deep, their absolute energies being approximately −3600 kcal/mol (4f_7/2_) and −3720 kcal/mol (4f_5/2_) [33]. From a chemical point of view, they are considered inert and are included in the core potential. The 5d orbitals present a much higher energetic value: −650 kcal/mol (5d_5/2_) and −714 kcal/mol (5d_3/2_). However, they remain low in energy; back-donation or electron promotion from these orbitals was checked: they are directional and can mix efficiently with the ligand p orbitals, as is frequently observed in transition metal complexes [34]. The population of full 5d orbitals or empty 6d orbitals was checked through NBO analysis (from the MP2 calculations) for the whole series of complexes studied. The 5d orbitals are weakly depopulated and this depopulation seems to be independent of the ligand: there are 9.87 electrons in these orbitals in the [Pb(H)_3_]^−^ complex. The value is the same (9.87 electrons) in the [Pb(CN)_3_]^−^ complex and in the [Pb(OH_2_)_3_]^2+^ complex (9.89 electrons). Charge transfer towards the empty 6d is also very weak, from 0.05 electrons in [Pb(H)_3_]^−^ to only 0.02 electrons in the [Pb(OH_2_)_3_]^2+^ complex. The occupation of these orbitals almost does not differ from that of the isolated Pb^2+^ cation and is furthermore almost ligand-independent. In conclusion, these orbitals are not involved in the bonding and will not be discussed any further. The true valence orbitals are therefore the full 6s_1/2_ (−355 kcal/mol) and the empty 6p orbitals: −138 kcal/mol (6p_3/2_) and −172 kcal/mol (6p_1/2_). These will be the focus of our study.

These two sets of orbitals are the most important as the 6p will accept electrons from donating ligands, and the full 6s orbital, thanks to its diffuseness, will prevent the approach of the ligand from being too short. Their role has been discussed [11] and it has been shown that, in the presence of a ligand field, the external valence shell of the cation is distorted to allow close contact with the ligands. Therefore, at this point, the questions of the precise nature of the Lead(II) valence basin and of the concrete structural and dynamical consequences of its existence remain to be addressed.

### 3.1. Structures and Properties of Lead Complexes

Different sets of Pb(II) complexes were investigated to test the influence of several parameters on the geometries. First, we recalled the results for the [Pb(CO)_n_]^2+^ series (n = 1 to 6), whose topology will be explored further. Then, we compared the structures of [Pb(X)_3_]^−^ complexes (X = HO^−^, CN^−^, HS^−^, H^−^, Cl^−^) to understand the influence of the anion on the structure. Regarding the [Pb(CO)_n_]^2+^ series, we retrieved the results previously published [35]: from n = 1 to n = 3, the Pb-C distances are insensitive to n (see Table 1). We observed a small depopulation of the 6s orbital (less than 0.1 electrons) and a steady increase in the 6p electron population. However, it should be noticed that this 6s depopulation increases from n = 1 to n = 3 and is almost nil for higher coordination numbers. This could be linked to the presence of a close carbonyl that destabilizes the 6s orbital to ensure coordination. Starting from coordination number 4, the Pb-C distances are no longer identical. In the [Pb(CO)_4_]^2+^ complex, there are two short distances and two longer. The latter correspond to the two CO, being in the trans position, and the former to CO for which there are no ligands in the trans position. As the lengthening of the Pb-C distances diminishes the interactions, the 6s population is allowed to increase, whereas the 6p population exhibits a more modest increase as the long Pb-C distances do not favor ligand donation. The general shape of the complexes is a butterfly for n = 4, square-based pyramid for n = 5, and the lead cation is outside the square plane and a perfect octahedron for n = 6. Interestingly, even for n = 2, the structure deviates from the ideal 90° for the C-Pb-C angle—the value is smaller, despite the ligand–ligand repulsion, showing that it is not this interaction that governs the structure.

**Figure 1:**
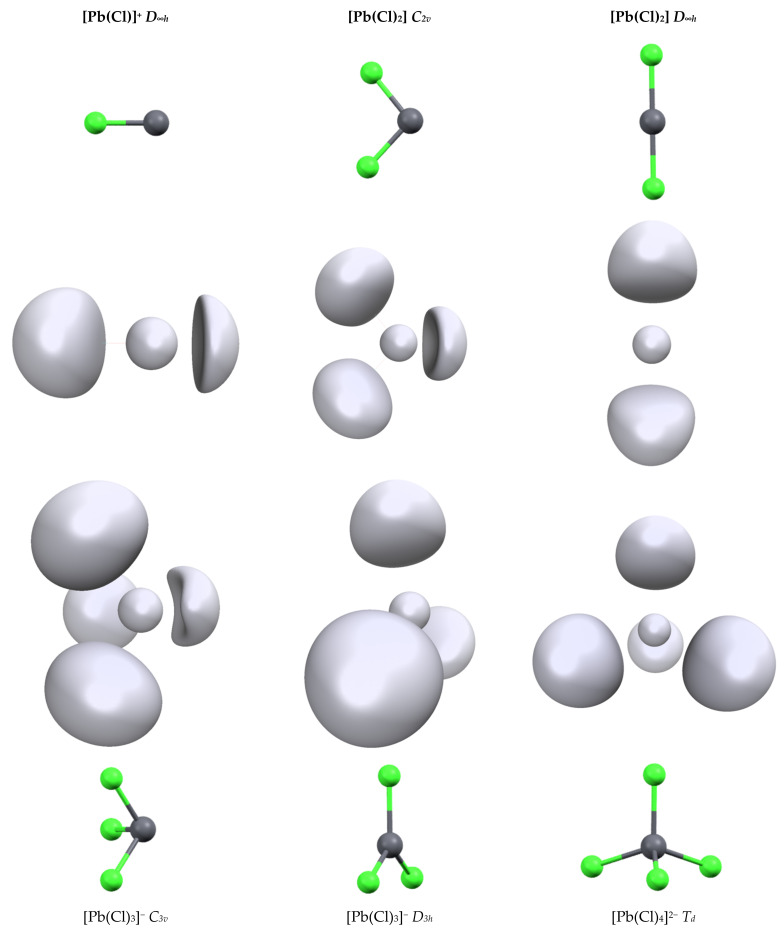
Structures and ELF function (η = 0.65) of the different [Pb(Cl)_n_]^q^ complexes.

The second series of complexes studied was [Pb(Cl)_n_]^q^ with n = 1 to 4 (Figure 1). All attempts to optimize them for a more highly coordinated structure led to ligand decoordination. The Pb-Cl distances are shorter than the Pb-C distances (see Table 2) and we can link this to the slightly larger 6s depopulation and 6p population. The geometry of the complexes has a strong effect on the bond length and orbital population. For [Pb(Cl)_2_] and [Pb(Cl)_3_]^−^ complexes, the structures of the highest symmetries (*D_∞h_* and *D_3h_*, respectively) lead to strong Pb-Cl distance lengthening (0.2 and 0.1 Å, respectively) compared to their lower-symmetry analogues. Simultaneously, the repopulation of the 6s shell and drop of the 6p shells is observed, although this is less pronounced in the [Pb(Cl)_3_]^−^ complex, highlighting again the strong trans effect in lead complexes. This is well illustrated by the characteristics of the [Pb(Cl)_4_]^2−^ complex. Contrary to the [Pb(CO)_4_]^2+^ complex, the structure of [Pb(Cl)_4_]^2−^ has the highest *T_d_* symmetry. The addition of a fourth chloride anion on the [Pb(Cl)_3_]^−^ complex leads to a strong increase in the Pb-Cl distance. In addition, a drop in the 6p shell population is observed despite the presence of an extra anion and also the repopulation of the 6s shell. The structure symmetry of the complex and the bond length increase. The diminishing overlap with the 6p orbitals lowers the constraint exertion on the 6s shell explaining these electronic evolutions.

Finally, we compared the structure of a series of Pb(L)_3_ complexes, either dicationic or monoanionic (Table 3). Surprisingly, no clear trends appeared in terms of the Pb-L bond lengths, L-Pb-L angles or orbital occupations. Indeed, the Pb-L distances are generally shorter if L is anionic (L = HO^−^) rather than if L is neutral (L = H_2_O). However, the Pb-L distances are almost identical for L = H_2_O and L = H_3_C^−^. Clearer observations were possible for the L-Pb-L angle. It is smaller for the neutral ligands, between 80° and 90°, than for the anions, which yield values around 90°–92°. These latter values can be understood as resulting from the maximized overlap with the cation 6p orbitals. However, the halide complexes deviate from this value, with the angle being larger than 97° and increasing with the halide size (from F^−^ to I^−^). The 6s orbital depopulation is stronger for the anionic ligand (up to 0.34 electron for [Pb(H)_3_]^−^) than for the neutral one, for which it is smaller than 0.1 electrons. The population of the 6p orbital is higher for the anionic ligand, due to their better σ-donation ability and 6s to 6p electron promotion compared to the neutral ligand. Inside the anionic ligand series, a soft ligand, according to HSAB theory, favors a greater 6p population (see halide series or compare L = HO^−^ and L = HS^−^).

**Figure 2:**
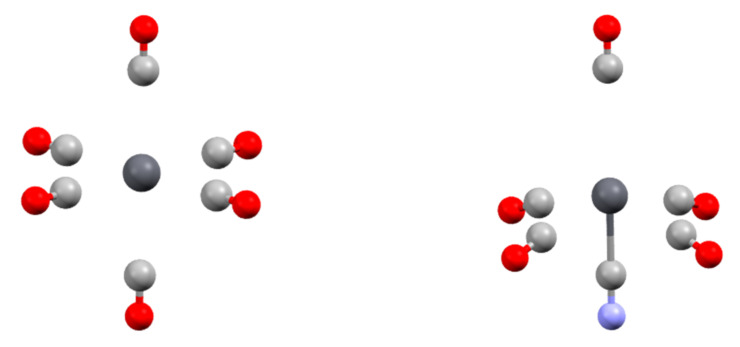
Structure of [Pb(CO)_6_]^2+^ (**left**) and [Pb(CO)_6_(CN)]^+^ (**right**) complexes.

One last point concerns the heterogeneous complexes. From the [Pb(CO)_n_]^2+^ and [Pb(Cl)_n_]^q^ series, we showed that placing ligands in trans positions leads to strong Pb-L distance lengthening. Stable structures can be optimized up to n = 6 for the CO series but up to only n = 4 for the Cl series. Under experimental conditions (biological environment and/or solvated media), the lead coordination sphere is generally heterogeneous with both neutral and anionic ligands. To explore the stability of such edifices, we progressively replaced one CO ligand within the optimized [Pb(CO)_6_]^2+^ structure by CN^−^ and reoptimized the structure. The structure of the [Pb(CN)(CO)_5_]^+^ complex was highly distorted compared to the [Pb(CO)_6_]^2+^ reference (Figure 2). The CO ligand trans to the cyano one was only weakly bonded to the complex, with a Pb-C distance of 3.643 Å. However, the carbonyls located in cis were also affected. If the bond length (2.828 Å) is similar to that in the hexacarbonyl complex, the angle between the cyano and the carbonyl ligand in cis is only 76.5°.

Two structures are possible for the [Pb(CN)_2_(CO)_4_] complex. A stable structure was found when the cyano ligands were in the trans position. In this case, the shape of the complex is that of an octahedron, with two short Pb-CN distances of 2.560 Å, though much larger than in the [Pb(CN)_3_]^−^ complex (Table 3), and four long Pb-CO distances (2.911 Å) larger than in the [Pb(CO)_6_]^2+^ complex. No stable structure was found when the cyano ligands were in the cis position. Indeed, the CO in the trans position with the cyano ligands were expelled from the complex. For [Pb(CN)_3_(CO)_3_]^−^, the situation was similar: all the carbonyl complexes were expelled from the complex. This suggests that in the gas phase, the trans effect is so strong that no neutral ligand can bind trans to an anionic ligand.

**Figure 3:**
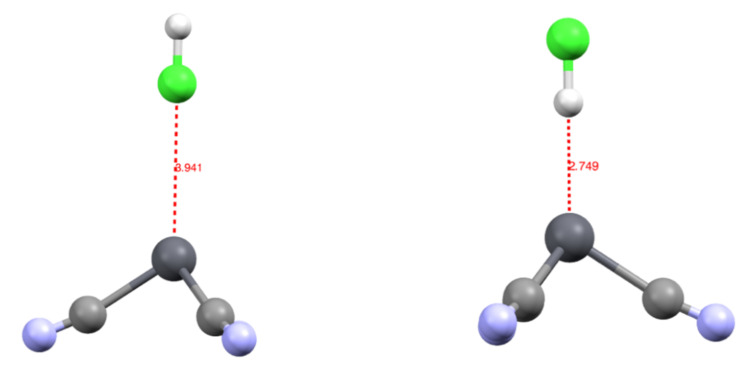
Structure of the [Pb(CN)_3_(ClH)]^−^ (**left**) and [Pb(CN)_3_(HCl)]^−^ (**right**) complexes with distances in Å.

We studied the complexation of an HCl molecule onto the [Pb(CN)_3_]^−^ complex. The approach was along the C_3_ axis of the complex, either through the chlorine [Pb(CN)_3_(ClH)]^−^ or hydrogen atom [Pb(CN)_3_(HCl)]^−^. The [Pb(CN)_3_(HCl)]^−^ was the most stable by 4.2 kcal/mol, with a Pb-H bond length of 2.749 Å. On the contrary, in the [Pb(CN)_3_(ClH)]^−^ complex, the Pb-Cl distance was 3.941 Å (Figure 3). This suggests that when searching for a new ligand to extract lead from media, not only does the direct coordination sphere need to be considered, but a positive pole may also play a role in complexing this coordination sphere.

### 3.2. Topology and Nature of Interactions

In a previous work, we have shown that the presence of the lead valence controls the structure of lead complexes. In particular, this basin explains the deactivation of a zinc finger metalloenzyme [13] or the structure of various complexes. We were able to correlate its properties (volume and density) to the nature of the ligand and also demonstrate some unexpected effects on the ligand, as with the thiocyanate ligand [14].

**Figure 4:**
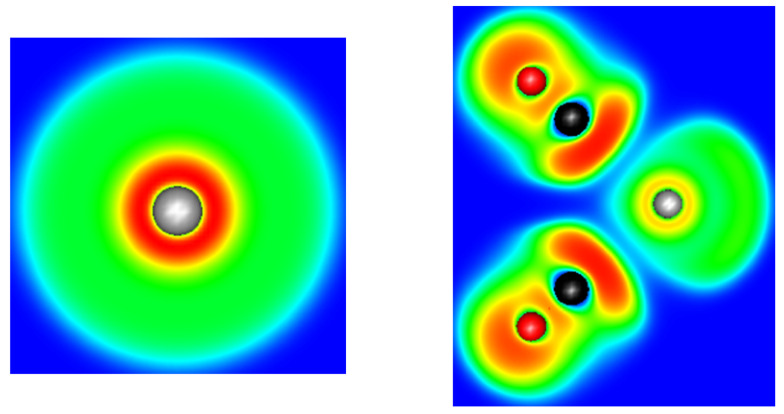
Cut plane of the ELF function of an isolated Pb^2+^ cation (**left**) and along the σ_v_ plane of the [Pb(CO)_2_]^2+^ complex (**right**). The range of the ELF functions varies from 0 (deep blue) to 1 (deep red).

The ELF basin structures of lead are strongly dependent on the cation coordination sphere. As we used a pseudopotential for our calculations, the computed ELF basins only contain the 5s, 5p, 5d, 6s and 6p shells. From the ELF point of view, this generates two kinds of ELF basins, a core basin and a valence basin. The electrons of the fifth shell and part of 6s generate the core one, the valence basin containing the rest of the 6s electrons and the population transferred in the 6p by ligand donation. This can be illustrated by the result performed on an isolated Pb^(II)^ cation: only 0.82 electrons are present in the valence basin, whereas 19.18 electrons are present in the core, which forms a perfect sphere around the cation (Figure 4). In the presence of a ligand field, both core and valence basins are affected. The core basin, which, for lead, represents the most external shell of core electrons in our computational conditions, splits itself into several fragments. This phenomenon is called subvalence and accounts for the cation polarizability [36]. We also observe a drop in the basin electronic population, which falls to 18.19 electrons in the [Pb(CN)_3_]^−^ complex. This is another illustration of the 6s to 6p electron promotion.

Upon ligand coordination, the valence basin is highly distorted. When a ligand binds to the cation, the basin population increases due to the 6p population. Furthermore, these orbitals being directional, they allow for the distortion of the basin, which is repelled to the opposite side of the ligand. In a multi-ligated complex, the valence basin tends to localize in the area of the weakest ligand field. This means that, for low coordination (n = 1 to 3), the ligand field will adopt a non-spherical (hemidirected) distribution and the valence basin attractor moves to the opposite side of the ligand. For higher coordination numbers, two kinds of ligand distribution are possible depending on the ligand nature. For n = 4, the complex [Pb(Cl)_4_]^2−^ adopts a spherical distribution (holodirected), whereas the complex [Pb(CO)_4_]^2+^ adopts an anisotropic structure (hemidirected). From the complexes studied and the results of Shimoni et al. [11], it seems that the more the ligand is a σ-donor, the higher the probability of the structure being holodirected. This is the case for the halide (except fluoride) or for the hydride and H_3_C^−^. To our knowledge, for n = 5, the complexes have only been observed in a hemidirected structure, forming an octahedron in which the valence basin localizes in the lacunary position. For n = 6, the complex is holodirected.

Table 2 illustrates the effect of the ligand field on the valence basin properties. We compared its volume and population in different structures of the [Pb(Cl)_2_] and [Pb(Cl)_3_]^−^ complexes. Moving from hemidirected to holodirected geometry leads to a significant drop in the volume and electronic population. For [Pb(Cl)_3_]^−^, the population decreases from 1.94 electrons (*C_3v_*) to 0.64 electrons (*D_3h_*). This evolution is partly due to the smaller available space into which the valence basin can expand. In the hemidirected *C_3v_* form, more than half of the coordination sphere is left available for the valence basin, whereas, in the holodirected *D_3h_*, the basin has to split into two halves on each side of the complex. This trend and the capability of the basin to expand explain the values of the L-Pb-L angle in Table 3. In the anionic complexes, this angle is slightly larger than the ideal 90° value, which maximizes the overlap between the 6p orbitals and the ligand. The small deviation may be due to the inter-ligand charge repulsion. On the contrary, for cationic complexes, this L-Pb-L angle is significantly lower (except for [Pb(NH_3_)_3_]^2+^). As there is less charge repulsion between the ligands, the pressure exerted by the valence basin on the ligand forces the reduction of this angle despite a lower overlap with the 6p orbitals.

**Figure 5:**
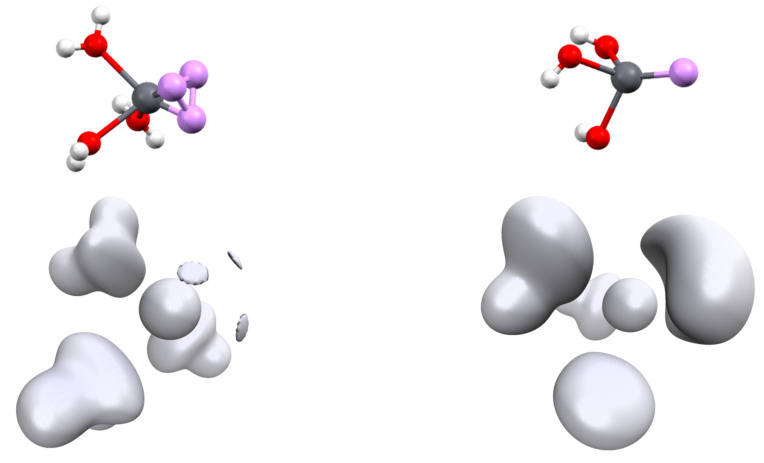
Position of the V(Pb) attractors (**top**) and associated ELF isosurface (η = 0.59, (**bottom**)) of [Pb(OH_2_)_3_]^2+^ (**left**) and [Pb(OH)_3_]^−^ (**right**) complexes.

Another parameter may be discussed, namely the position of the ELF attractor(s) and especially its or their distances with the lead cation. Indeed, for most of the complexes, there is only one Pb^2+^ valence basin. The structure of the complexes is a tetrahedron, with V(Pb) occupying one of the summits and the cation being at the center (Figure 5, top right). However, for the smallest donor ligand, namely OH_2_, NH_3_ or HCN, the valence basin is split into three (Figure 5, top left). For these compounds, there are three valence basins. The description of Pb^2+^ valence basins cannot be easily reduced to a hybridized sp^3^ lone pair—it is much more versatile.

In Table 1 and Table 2, it is shown that this attractor goes further from the cation up to n = 3; then, its distance does not evolve any longer in the hemidirected structures. This is the effect of the Pb^2+^ polarization due to the ligand field. This is confirmed in Table 3, in which d(Pb), the ELF attractor distance from the lead cation, is correlated to the σ-donating forces of the ligand. This force can be assimilated to N(Pb), the population of V(Pb). Plotting d(Pb) vs. N(Pb) leads to a linear arrangement (Figure S1, Supplementary Materials) of the ligand: the more donations to lead there are, the further V(Pb) is repealed.

**Figure 6:**
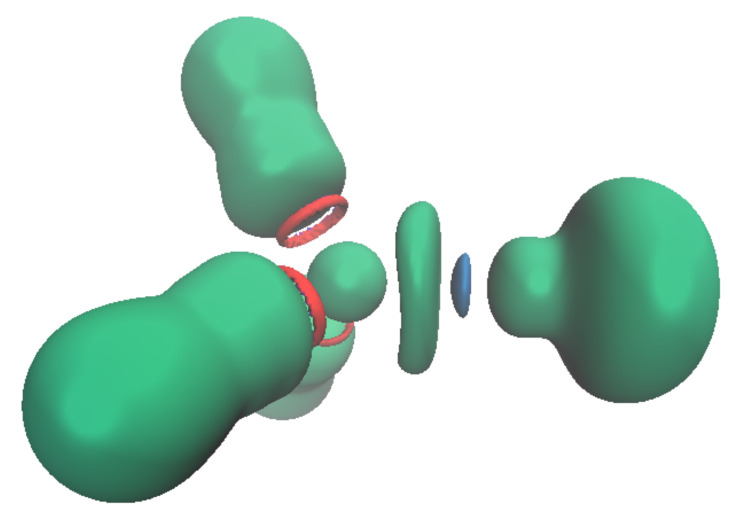
Topology of the [Pb(CN)_3_(HCl)]^−^ complex. The ELF basin (isovalue η = 0.6) is shown in green; the NCI (isovalue η = 0.552) interactions are shown in red and blue. The areas in red correspond to steric repulsion and that in blue to electrostatic attraction.

This valence basin arising from the polarization of the Pb^2+^ external electronic shell translates to an excess of electronic density trans to the more σ-donor ligand. In consequence, there will be a discrimination regarding the ligand that can bind to the cation: the less donating will be expelled from the coordination sphere when being in trans to a stronger donor group. This is illustrated by the [Pb(CO)_3_(CN)_3_]^−^ complex, in which the neutral CO are unstable in the first coordination sphere. This valence basin will also have further consequences on the organization of the cation’s second coordination sphere. As already mentioned, this valence basin forms an electronic shield, preventing further coordination at this position. However, we attempted to approach an HCl molecule on the cation either through the chlorine atom or through the hydrogen atoms. The structure with the hydrogen atom pointing toward the lead (Pb-H distance at 2.781Å) was more stable by 4.3 kcal/mol than the conformer (Pb-Cl distance at 3.941 Å). The NCI analysis performed on the optimized structures (Figure 6) showed the presence of an attractive electrostatic force between the ELF valence basin of the cation and the hydrogen valence basin of the HCl. This means that the cation valence basin occupying the complex lacuna can be described as a negative pole that is able to interact with positively charged fragments, mimicking a sort of hydrogen bond, as suggested by Hancock [37].

Furthermore, it should be noticed that all the structures discussed until now have been optimized at the MP2 level of theory. Optimization with the B3LYP functional without dispersion correction of the [Pb(CN)_3_(ClH)]^−^ complex failed to find any minima and led to the decoordination of the complex. On the contrary, optimization with the ωB97XD functional led to results close to the MP2 ones. The [Pb(CN)_3_(HCl)]^−^ structure (Pb-H distance of 2.884 Å) is more stable than the [Pb(CN)_3_(ClH)]^−^ one (Pb-Cl distance of 4.324 Å) by 2 kcal/mol. It will be critical in lead complexes to include dispersion corrections (e.g., through Grimme’s correction [38]) when the lead valence basin may interact with the second coordination sphere ligand. We further explored the importance of the population of the Pb^2+^ valence basin by complexing the HCl on the [Pb(CO)_3_]^2+^ complex with the ωB97XD functional. The situation was completely different. In this case, no minima were found for the [Pb(CO)_3_(HCl)]^2+^ complex. When the hydrogen pointed toward the lead, the HCl molecule was expelled from the complex. On the contrary, there were minima for the [Pb(CO)_3_(ClH)]^2+^ complex, with a Pb-Cl distance of 3.041 Å, much shorter than in the [Pb(CN)_3_(ClH)]^−^ complex. This suggests that the structuration of the solvent (the orientation of the molecule) around the valence basin of Pb^2+^ will be dependent on the ligand held by the cation.

### 3.3. Interconversion Barriers

**Figure 7:**
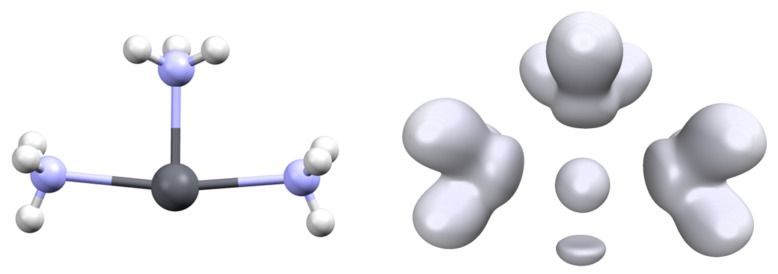
Structure (T shape) of the transition state for the interconversion of the [Pb(NH_3_)_3_]^2+^ complex (**left**) and associated ELF analysis ((**right**), isovalue η = 0.6).

The hemidirected nature of the [Pb(L)_3_]^q^ complexes (isostructural to NH_3_) implies the existence of two conformers separated by an interconversion barrier. For some complexes, we computed the transition state between the two minima and determined this barrier. These calculations were performed with GAUSSIAN. Surprisingly, the transition state for the interconversion of most complexes is not of *D_3h_* but adopts a T shape (Figure 7). Consequently, the three Pb-L distances are not identical, with one short and two long for the two ligands in the trans position. Only the mono-atomic ligand (H^−^, F^−^ and Cl^−^) exhibits a *D_3h_* transition state.

The interconversion barrier is strongly dependent on the ligand (Table 4). The main point seems to be the σ-donor ability. The strongest σ-donors [Pb(H)_3_]^−^ and [Pb(Me)_3_]^−^ have the highest barrier, more than 40 kcal/mol, meaning that the interconversion is not thermally accessible at room temperature. On the contrary, poor σ-donor ligands such as water have a very low barrier and do not have any fixed configuration. Most of the ligands exhibit moderate values, between 15 and 30 kcal/mol. This suggests the possibility of synthesizing optically active organolead complexes.

Again, the value of this barrier reflects the flexibility/rigidity of the lead valence basin. Formally, the interconversion consists of the migration of the valence basin from one side complex plane to the other side. In a *D_3h_* symmetry transition state, this leads to a drop in the interaction between the cation and the ligand (see the *C_3v_* and *D_3h_* structure of [Pb(Cl)_3_]^−^, Table 2). The T-shape structure for the TS allows the weakening of only two of the lead–ligand interactions. The geometry can also be a square planar complex in which one of the positions is occupied by the valence basin (Figure 3).

**Figure 8:**
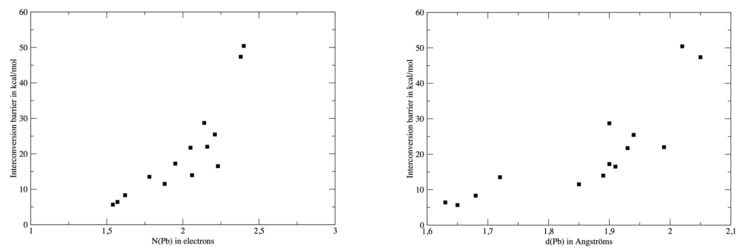
Values of the interconversion barrier against N(Pb), (**left**) or d(Pb) (**right**). Values were computed at the DFT level of theory with the ωB97XD functional.

To further explore the relationship between the interconversion barriers (Table 4) and the topological properties of the complexes, we plotted the barrier values against N(Pb), the electronic population of V(Pb) or d(Pb) (Figure 8). For consistency, the topological analysis was re-performed on the ωB97XD wavefunction and the barrier values computed at the same level were used for the analysis. The relationship between d(Pb) and the barrier is quite poor, though a high barrier value is roughly associated with a larger d(Pb). However, three groups of ligands can be identified. The neutral ligands (OH_2_, HCN, CO or NH_3_) are associated with low barriers and short d(Pb) distances. A second group consisting of H^−^ and H_3_C^−^ exhibits both a high barrier and large d(Pb). The other anions are intermediate, but a significant gap (roughly 20 kcal/mol) distinguishes them from the second group. The link between N(Pb) and the barrier is more significant, although the three groups already identified are still valid. This supports our analysis of a direct link between the σ-donor capacity of the ligand, the topology of the valence basin of lead and the interconversion barrier.

## 4. Conclusions

In this contribution, we have studied the nature of the lead valence basins and, especially, what we refer to as the lone pair in previous studies. Through molecular orbital analysis, NBO and NPA analyses and the study of the molecular contribution to the ELF basin, we have shown that the structures of the lead complexes are only governed by the 6s and 6p subshells. No involvement of the d orbitals has been observed: the depopulation of the 5d orbitals is minimal and does not seem to be ligand-dependent according to NBO analysis; in addition, the charge transfer to the empty 6d is negligible. The 5d subshell is too low in energy and too contracted to interact with the valence orbital of the ligands. Similarly, the 6d orbitals are too high in energy and do not contribute. The complexation of Pb^2+^ can be explained through interaction with the 6s^2^ electrons and the accepting 6p orbitals.

The number of ELF attractors depends on the nature of the Pb^2+^ ligands: the less donating ones (essentially the neutral ones as OH_2_) generate three attractors, whereas the stronger σ-donors generate only one attractor. It is thus difficult to reduce the presence of the valence basin to a hybridized sp^3^ lone pair, due to the weak contribution of the p orbitals to this lone pair, as already shown by Shimoni [11].

The existence of this valence basin has structural and dynamic consequences. Generated by the promotion of 6s electrons to the 6p orbitals and by the σ-donation from the ligand, its volume and population will increase with the strength of the Pb–L interaction. Simultaneously, the distribution of the ligand will be governed by the trans effect. This means that if a strong σ-donor ligand binds to Pb^2+^, the position in trans (considering an octahedron) will be destabilized, as we observed on our attempt to optimize the [Pb(CN)_3_(CO)_3_]^−^ complex. Moreover, this indicates that a second σ-donor ligand will bind preferentially in the cis position. This is due to the polarization of the valence basin of lead, which moves trans to the ligand and strongly weakens the electrophilicity of the cation in this position in the sense that the electronic density locally increases. In [Pb(L)_3_]^q^ complexes, this can even lead to local nucleophilicity, as illustrated by the preference for an interaction with the H atom of HCl of the [Pb(CN)_3_]^−^ complex. This paves the way towards the design of new families of specific ligands for Pb^2+^ having a positive pole in their structure.

Other consequences concern the dynamical effect of this lone pair. To our knowledge, no simulation has been conducted on [Pb(L)_4_]^2−^ complexes. If energy minima have been found, the question of their dynamic stability has still not been answered. Indeed, the strong destabilization of the two anions located in the trans position paves the way towards fast exchange with solvent molecules, to form a [Pb(L)_3_]^−^ complex plus a solvated L^−^. Another consequence is the configuration stability of [Pb(L)_3_]^−^ complexes. If the cation binds to three different ligands, especially organic ones, the interconversion barrier may be high enough to block the configuration, presenting the opportunity for the synthesis of enantiopure compounds.

## Figures and Tables

**Table 1:** Properties of the [Pb(CO)_n_]^2+^ complexes. Pb-C bond lengths in Angstroms. C-Pb-C angle in degrees. Electronic population of the 6s and 6p shells of lead from NBO analysis. Volume (ω(Pb) in Å^3^), population (N(Pb) in electrons) of lead valence basin and distance (d(Pb) in Å) of the V(Pb) ELF attractor and cation.

	Pb-C	C-Pb-C	6s	6p	V(Pb)		
					ω(Pb)	N(Pb)	d(Pb)
[Pb(CO)]^2+^	2.612	NC	1.94	0.17	165.6	1.25	1.583
[Pb(CO)_2_]^2+^	2.627	83.1	1.93	0.35	160.6	1.42	1.587
[Pb(CO)_3_]^2+^	2.635	82.1	1.91	0.58	156.0	1.52	1.620
[Pb(CO)_4_]^2+^	2.635 2.777	81.5 150.0	1.95	0.59	135.3	1.44	1.616
[Pb(CO)_5_]^2+^	2.635 2.796	76.3 86.6 152.5	1.95	0.71	106.5	1.27	1.618
[Pb(CO)_6_]^2+^	2.845	90.0	1.98	0.77			

**Table 2:** Properties of the [Pb(Cl)_n_]^q^ complexes. Pb-Cl bond lengths in Angstroms. Cl-Pb-Cl angle in degrees. Electronic population of the 6s and 6p shells of lead from NBO analysis. Volume (ω(Pb) in Å^3^), population (N(Pb) in electrons) of lead valence basin and distance (d(Pb) in Å) of the V(Pb) ELF attractor and cation.

	Pb-Cl	Cl-Pb-Cl	6s	6p	V(Pb)		
					ω(Pb)	N(Pb)	d(Pb)
[Pb(Cl)]^+^	2.348	NC	1.91	0.55	216.4	1.78	1.707
[Pb(Cl_2_] (*C_2v_*)	2.449	99.3	1.87	0.89	213.2	1.95	1.787
[Pb(Cl_2_] (*D_∞h_*)	2.566	180	1.99	0.39	182.7	1.32	1.401
[Pb(Cl)_3_]^−^ (*C_3v_*)	2.560	99.8	1.84	0.96	186.6	1.94	1.837
[Pb(Cl)_3_]^−^ (*D_3h_*)	2.660	120.0	1.93	0.72	79.48	0.64	1.439
[Pb(Cl)_4_]^2−^ (*T_d_*)	2.781	109.5	1.93	0.74	56.9	0.62	1.225

**Table 3:** Properties of [Pb(L)_3_]^q^ complexes. Pb-L bond length in Angstroms. L-Pb-L angle in degrees. Electronic population of the 6s and 6p shells of lead from NBO analysis. Volume (ω(Pb) in Å^3^), population (N(Pb) in electrons) of lead valence basin and distance (d(Pb) in Å) of the V(Pb) ELF attractor and cation.

	Pb-L	L-Pb-L	6s	6p	V(Pb)		
					ω(Pb)	N(Pb)	d(Pb)
[Pb(H)_3_]^−^	1.848	91.2	1.66	2.45	286.5	2.51	1.966
[Pb(Me)_3_]^−^	2.328	90.8	1.68	1.80	282.9	2.48	1.988
[Pb(F)_3_]^−^	2.115	97.1	1.78	0.61	210.9	2.01	1.898
[Pb(Cl)_3_]^−^	2.560	99.8	1.84	0.96	186.6	1.94	1.837
[Pb(Br)_3_]^−^	2.719	100.6	1.87	1.15	183.5	1.96	1.810
[Pb(I)_3_]^−^	2.911	100.8	1.88	1.34	182.6	2.04	1.787
[Pb(CN)_3_]^−^	2.306	91.9	1.73	1.50	212.5	2.19	1.882
[Pb(OH)_3_]^−^	2.176	91.8	1.78	0.96	236.7	2.13	1.935
[Pb(SH)_3_]^−^	2.636	89.9	1.80	1.34	214.4	2.17	1.859
[Pb(HCN)_3_]^2+^	2.415	84.7	1.89	0.43	161.8	1.65	1.682
[Pb(CO)_3_]^2+^	2.635	82.1	1.91	0.58	156.0	1.52	1.620
[Pb(OH_2_)_3_]^2+^	2.374	83.8	1.91	0.31	158.9	1.58	1.638
[Pb(NH_3_)_3_]^2+^	2.451	90.0	1.89	0.61	172.9	1.82	1.690

**Table 4:** General shape and interconversion barriers (in kcal/mol) with B3LYP and ωB97XD functionals.

Complex	Structure	∆G (B3LYP)	∆G (ωB97XD)
[Pb(H)_3_]^−^	*D_3h_*	53.1	50.4
[Pb(Me)_3_]^−^	T shape	44.8	47.4
[Pb(CN)_3_]^−^	T shape	27.0	28.7
[Pb(OH)_3_]^−^	T shape	23.6	25.4
[Pb(NH_2_)_3_]^−^	T shape	20.7	22.0
[Pb(OMe)_3_]^−^	T shape	19.6	21.7
[Pb(F)_3_]^−^	*D_3h_*	16.0	17.2
[Pb(SMe)_3_]^−^	T shape	15.2	16.5
[Pb(SH)_3_]^−^	T shape	14.4	14.0
[Pb(NH_3_)_3_]^2+^	T shape	12.2	13.5
[Pb(Cl)_3_]^−^	*D_3h_*	10.7	11.5
[Pb(HCN)_3_]^2+^	T shape	7.9	8.3
[Pb(CO)_3_]^2+^	T shape	6.4	6.4
[Pb(OH_2_)_3_]^2+^	T shape	5.2	5.7

## Data Availability

Not applicable.

## Supplementary Materials

The following are available online, Figure S1: Distance between the ELF attractor of the lead valence basin in function of the population of V(Pb), data extracted from Table 3, Table S1: Pb-L distances in Angströms and L-Pb-L angles in degrees of the complexes optimized with the different methods.
