# Supplementary material for: On the Quantum Chemical Nature of Lead(II) “Lone Pair”
Source: Molecules. 2021 Dec 22;27(1):27. doi: 10.3390/molecules27010027 (PMC8746439; doi:10.3390/molecules27010027)
Supplement: Supplementary file 1 [file molecules-27-00027-s001.zip › molecules-1249768-supplementary.pdf]

molecules

Supplementary Materials

On the Nature of Lead(II) "Lone Pair"

Christophe Goulaouen [1,*] and Jean-Philip Piquemal [2,3,4,*]

1 Laboratoire de Chimie Quantique, UMR7177 CNRS et Université de Strasbourg, 67000 Strasbourg, France
2 Laboratoire de Chimie Théorique, Sorbonne Université, UMR7616 CNRS, 75005 Paris, France
3 Institut Universitaire de France, 75005 Paris, France
4 Department of Biomedical Engineering, The University of Texas at Austin, Austin, TX 78712, USA
* Correspondence: gourlaouen@unistra.fr (C.G.); jean-philip.piquemal@sorbonne-universite.fr (J.-P.P.)

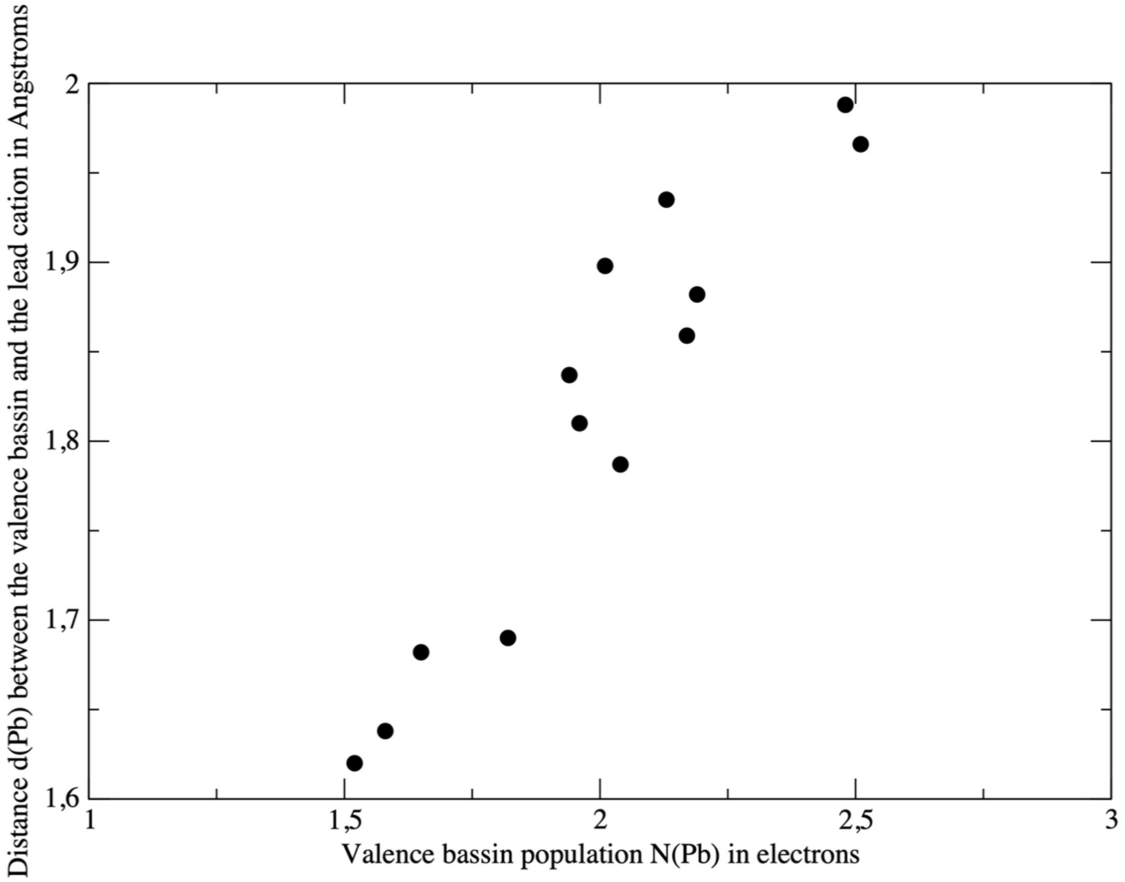

Figure S1. Distance between the ELF attractor of the lead valence basin in function of the population of V(Pb), data extracted from Table 3.

Table S1. Pb-L distances in Angströms and L-Pb-L angles in degrees of the complexes optimized with the different methods.

	**Turbomole**		**GAUSSIAN**			
	**MP2**		**B3LYP**		**wB97XD**	
	**Pb-L**	**L-Pb-L**	**Pb-L**	**L-Pb-L**	**Pb-L**	**L-Pb-L**
$[Pb(H)_3]^-$	1.848	91.2	1.894	91.8	1.881	91.9
$[Pb(Me)_3]^-$	2.328	90.8	2.386	93.1	2.348	92.1
$[Pb(F)_3]^-$	2.115	97.1	2.142	97.7	2.125	96.9
$[Pb(Cl)_3]^-$	2.560	99.8	2.613	100.8	2.587	99.5
$[Pb(Br)_3]^-$	2.719	100.6	2.776	102.2	2.742	100.5
$[Pb(I)_3]^-$	2.911	100.8	2.991	103.2	2.956	101.2
$[Pb(CN)_3]^-$	2.306	91.9	2.358	93.6	2.337	92.7
$[Pb(OH)_3]^-$	2.176	91.8	2.207	93.4	2.188	92.6
$[Pb(SH)_3]^-$	2.619	94.6	2.690	97.2	2.652	96.0
$[Pb(HCN)_3]^{2+}$	2.415	84.7	2.457	89.0	2.447	87.0
$[Pb(CO)_3]^{2+}$	2.635	81.9	2.702	86.7	2.683	83.3
$[Pb(OH_2)_3]^{2+}$	2.374	83.8	2.406	88.2	2.400	85.4
$[Pb(NH_3)_3]^{2+}$	2.451	90.0	2.503	92.3	2.480	90.3
